# Sickle Cell Hepatopathy With Acute Hepatic Sequestration and Extreme Hyperbilirubinemia

**DOI:** 10.7759/cureus.84888

**Published:** 2025-05-27

**Authors:** Usman Bin Hameed, Joel Karsten, Fady Banno, Brian Nadeau

**Affiliations:** 1 Internal Medicine, Corewell Health William Beaumont University Hospital, Royal Oak, USA; 2 Internal Medicine, Oakland University William Beaumont School of Medicine, Auburn Hills, USA; 3 Gastroenterology, Corewell Health William Beaumont University Hospital, Royal Oak, USA; 4 Hepatology, Corewell Health William Beaumont University Hospital, Royal Oak, USA

**Keywords:** acute hepatic sequestration, acute intrahepatic cholestasis, acute sickle hepatic crisis, double heterozygous hbs and beta thalassemia trait, exchange transfusion, extreme hyperbilirubinemia, hepatic complication of sickle cell disease, sickle cell crisis, vaso-occlusive crisis

## Abstract

Sickle cell disease (SCD) is a genetic disorder characterized by chronic hemolytic anemia and multi-organ dysfunction. The liver is frequently affected in SCD, with complications spanning from mild hyperbilirubinemia to acute liver failure. Acute hepatic sequestration (AHS), a rare but serious manifestation of sickle cell hepatopathy, can rapidly progress to multi-organ dysfunction syndrome (MODS). Cases with extreme hyperbilirubinemia (bilirubin > 20 mg/dL) remain exceptionally uncommon.

A 19-year-old female patient with SCD presented with nausea, vomiting, generalized pain, dysuria, and suprapubic pain. On presentation, her vital signs were stable except for tachycardia (139 beats per minute (bpm)). Physical examination revealed scleral icterus, conjunctival pallor, and right upper quadrant (RUQ) tenderness. Initial laboratory results showed leukocytosis (white blood cell (WBC): 22.7 bil/L), hemoglobin (Hb) of 7.5 g/dL (baseline: 8.5 g/dL), and platelet (PLT) of 178 bil/L. Lactate dehydrogenase (LDH) was at 734 U/L. Liver function tests showed alkaline phosphatase (ALP) at 238 IU/L, aspartate transaminase (AST) at 52 U/L, alanine transaminase (ALT) at 49 U/L, and total bilirubin at 13.4 mg/dL. Abdominal ultrasound showed hepatomegaly (liver span of 19 cm) and gallstones without signs of cholecystitis. Blood cultures (2/2) tested positive for *Escherichia coli* (*E. coli*). Initial management included intravenous (IV) fluids, pain control, IV antibiotics, and two units of packed red blood cells (RBCs). Despite treatment, total bilirubin rose to 35.1 mg/dL with direct bilirubin at >15 mg/dL, hemoglobin dropped to 5.5 g/dL, and platelet count decreased to 124 bil/L. An elevated reticulocyte count (295 × 10⁹/L) and percentage (10.47%) confirmed hepatic sequestration. Exchange transfusion improved the percentage of hemoglobin S (HbS) from 58.9% to 23.4%, decreased bilirubin levels to 12.1 mg/dL, and alleviated symptoms.

AHS is rare, occurring in about 1.5% of SCD patients. Extreme hyperbilirubinemia in this case (total bilirubin of 35.1 mg/dL) was more severe than usually seen in AHS, reflecting the severity of the clinical presentation. Differentiating AHS from other hepatic complications, such as acute sickle cell hepatic crisis (ASCHC) and acute intrahepatic cholestasis (AIC), is crucial due to differing management approaches. While ASCHC often responds to supportive care, AHS may require urgent transfusion, and AIC might necessitate invasive interventions, including liver transplantation. Exchange transfusion is effective in AHS by rapidly reducing HbS levels, improving hepatic function, and preventing progression to MODS. Given that AHS is a rare but critical complication of SCD, this case highlights the importance of prompt recognition, a high index of suspicion, and early intervention to optimize outcomes in severe SCD-related hepatic complications.

## Introduction

Sickle cell disease (SCD) is a genetic disorder caused by mutations in the β-globin gene, resulting in chronic hemolytic anemia, characterized by premature destruction of red blood cells (RBCs), and multi-organ dysfunction [1]. In SCD, abnormal hemoglobin S (HbS) polymerizes under stress, deforming RBCs into rigid, sickled shapes. These cells obstruct small vessels, causing vaso-occlusive crises, typically affecting lungs, bones, spleen, or kidneys, but potentially involving any organ [1].

Hepatic complications, collectively termed sickle cell hepatopathy, range from mild hyperbilirubinemia to acute liver failure or hemorrhage from coagulopathy [2]. Three acute hepatopathies are recognized: acute sickle cell hepatic crisis (ASCHC), marked by transient liver enzyme and bilirubin elevation; acute hepatic sequestration (AHS), involving rapid pooling of sickled RBCs in the liver, causing severe anemia and hepatomegaly; and acute intrahepatic cholestasis (AIC), characterized by profound cholestasis and extreme hyperbilirubinemia [2].

AHS is rare yet severe, often rapidly progressing to multi-organ dysfunction syndrome (MODS), a leading cause of mortality in SCD. AHS with extreme hyperbilirubinemia (bilirubin > 20 mg/dL) is exceptionally uncommon, underscoring the need for timely recognition and management.

This case describes AHS with extreme hyperbilirubinemia in a young female patient.

## Case presentation

A 19-year-old female patient with SCD (hemoglobin S/beta (β) 0 thalassemia) was admitted to the hospital for nausea, vomiting, generalized pain, and syncopal episodes, as well as dysuria and suprapubic pain over the last week. On presentation, her vitals were stable except for tachycardia at 139 beats per minute (bpm). Physical examination revealed scleral icterus, conjunctival pallor, and abdominal tenderness in the right upper quadrant (RUQ). Laboratory results (Tables 1-3) showed white blood cell (WBC) count of 22.7 bil/L (reference range: 3.3-10.7 bil/L), hemoglobin (Hb) of 7.5 g/dL (reference range: 12.1-15.0 g/dL, patient's baseline: 8.5 g/dL), platelet count of 178 bil/L (reference range: 150-400 bil/L), creatinine of 1.41 mg/dL (reference range: 0.50-1.10 mg/dL), and lactate dehydrogenase (LDH) of 734 U/L (reference range: 100-240 IU/L). Liver tests revealed an elevated alkaline phosphatase (ALP) of 238 IU/L (reference range: 33-120 IU/L), aspartate transaminase (AST) of 52 U/L (reference range: <35 IU/L), alanine transaminase (ALT) of 49 IU/L (reference range: 8-37 IU/L), and total bilirubin of 13.4 mg/dL (reference range: 0.3-1.2 mg/dL).

**Table 1:** Complete blood count Trends showing worsening during hepatic sequestration and subsequent improvement post-treatment. WBC: white blood cell, RBC: red blood cell, MCV: mean corpuscular volume, MCH: mean corpuscular hemoglobin, MCHC: mean corpuscular hemoglobin concentration, RDW-CV: red cell distribution width-coefficient of variation, HbS: hemoglobin S

Laboratory results	Admission	Hepatic sequestration	Post-treatment	Reference range
Hemoglobin	7.5 g/dL	5.5 g/dL	8.7 g/dL	12.1-15.0 g/dL
WBC	22.7 bil/L	28.3 bil/L	17.2 bil/L	3.3-10.7 bil/L
RBC	2.72 tril/L	2.02 tril/L	3.04 tril/L	3.87-5.08 tril/L
Hematocrit	21.4%	15.6%	24.1%	35.4%-44.2%
MCV	79 fL	77 fL	79 fL	80-100 fL
MCH	28 pg	27 pg	29 pg	28-33 pg
MCHC	35 g/dL	35 g/dL	36 g/dL	32-35 g/dL
RDW-CV	18%	17%	19%	12%-15%
Platelets	178 bil/L	124 bil/L	125 bil/L	150-400 bil/L
Hemoglobin variant (HbS)	-	58.9%	23.4%	<0.0%

**Table 2:** Hepatic function panel Trends highlighting marked hyperbilirubinemia during hepatic sequestration with improvement following treatment.

Laboratory results	Admission	Hepatic sequestration	Post-treatment	Reference range
Alkaline phosphatase	238 U/L	208 U/L	193 U/L	33-120 U/L
Aspartate aminotransferase	52 U/L	39 U/L	33 U/L	<35 U/L
Alanine aminotransferase	49 U/L	35 U/L	24 U/L	8-37 U/L
Bilirubin (total)	13.4 mg/dL	35.5 mg/dL	12.1 mg/dL	0.3-1.2 mg/dL
Bilirubin (direct)	4.9 mg/dL	>15.0 mg/dL	9.1 mg/dL	0.0-0.4 mg/dL

**Table 3:** Basic metabolic profile Admission laboratory results showing metabolic acidosis, mild renal dysfunction, and elevated LDH, suggestive of hemolysis and hepatic involvement. LDH: lactate dehydrogenase, eGFR: estimated glomerular filtration rate

Laboratory results	Admission	Reference range
Sodium	137 mmol/L	135-145 mmol/L
Potassium	3.8 mmol/L	3.5-5.2 mmol/L
Chloride	106 mmol/L	98-111 mmol/L
Bicarbonate	14 mmol/L	20-29 mmol/L
Anion gap	17	5-17
Glucose	113 mg/dL	60-99 mg/dL
Blood urea nitrogen	30 mg/dL	7-25 mg/dL
Creatinine	1.41 mg/dL	0.50-1.10 mg/dL
eGFR	55 mL/minute/1.73 m^2^	>90 mL/minute/1.73 m^2^
Calcium	9.5 mg/dL	8.5-10.5 mg/dL
Protein (total)	7.2 g/dL	6.4-8.3 g/dL
Lactate dehydrogenase	734 U/L	100-240 U/L

Additionally, both blood cultures (2/2) were positive for *Escherichia coli* (*E. coli*), with urinalysis showing negative nitrates but positive leukocyte esterase (Table 4). However, urine cultures remained negative. The patient was started on cefepime 2 g for the treatment of bacteremia.

**Table 4:** Urinalysis Urinalysis on admission showing pyuria, hematuria, proteinuria, and bacteriuria. RBC: red blood cell, WBC: white blood cell, hpf: high-powered field, lpf: low-powered field

Laboratory results	Admission	Reference range
Color	Amber	Yellow
Clarity	Cloudy	Clear
Glucose	Negative	Negative (mg/dL)
Bilirubin	Negative	Negative
Ketones	Negative	Negative (mg/dL)
Specific gravity	1.010	1.005-1.030
Blood	2+	Negative
pH	5.0	5.0-8.0
Protein	100	Negative (mg/dL)
Urobilinogen	≥4.0 mg/dL	<2.0 mg/dL
Nitrites	Negative	Negative
Leukocyte esterase	3+	Negative
RBC	6-10	0-2 (negative)/hpf
WBC	>50	0-5 (negative)/hpf
Epithelial, squamous	1-5	Negative/lpf
Casts, hyaline	0-2	0-2 (negative)/lpf
Bacteria	1+	Negative/hpf

Computed tomography angiography was negative for pulmonary embolism. Given the hypothesis of hepatobiliary-origin bacteremia, coupled with worsening RUQ pain and elevated liver function tests, an abdominal ultrasound and magnetic resonance imaging (MRI)/magnetic resonance cholangiopancreatography (MRCP) (Video 1) was obtained. It revealed gallstones and an enlarged liver (19 cm) without signs of cholecystitis or cholangitis.

**Video 1:** MRCP T2 axial MRCP shows cholelithiasis with sludge, without evidence of cholecystitis or choledocholithiasis; other abdominal organs are unremarkable. MRCP: magnetic resonance cholangiopancreatography

Initial management included intravenous (IV) fluid resuscitation, pain control, and two units of packed RBC transfusions. Despite initial treatment, she continued to clinically decline, with worsening jaundice, drowsiness, and abdominal pain. Her bilirubin levels increased to 35.1 mg/dL with direct bilirubin > 15 mg/dL. Coupled with the patient's hemoglobin dropping to 5.5 g/dL, platelet count to 124 bil/L, and an elevated reticulocyte count of 295 × 10⁹/L (reference range: 21-100 × 10⁹/L) with a reticulocyte percentage of 10.47% (reference range: 0.48%-2.22%), hepatic sequestration was suspected.

An exchange blood transfusion was initiated. Following the transfusion, HbS percentage improved from 58.9% to 23.4% (normal values: <0.0%), leading to improved bilirubin levels at 12.1 mg/dL and resolution of abdominal pain. The patient's condition significantly improved by discharge with stable vitals and a recommendation for close follow-up.

## Discussion

Sickle cell crisis can be triggered by various factors, including dehydration, hypoxia, cold weather, or inflammation. In this case, bacteremia likely triggered the sickle cell crisis, potentially from a urinary source. Although urine cultures were negative, the presence of leukocyte esterase on urinalysis, *E. coli* bacteremia, and lack of alternative infection sources support a urinary tract origin. Negative urine cultures may result from prior antibiotic therapy, transient bacteriuria, or bacterial counts below detection thresholds. Inflammation leads to hypoxia, which prompts the polymerization of HbS within RBCs, causing them to assume their characteristic sickle shape. These sickled RBCs aggregate and obstruct small blood vessels, resulting in ischemia in downstream tissues [3,4]. While this process can affect multiple organs, the liver is particularly vulnerable due to its dense network of small vessels, contributing to hepatic involvement in 10%-40% of sickle cell crises [2].

ASCHC is the most common hepatic complication in SCD, occurring in up to 10% of patients [5]. It is defined by sinusoidal occlusion and infarction, potentially leading to centrilobular necrosis. Clinical presentation includes fever, RUQ pain, jaundice, and tender hepatomegaly. For laboratory values, transaminases can vary from normal to approximately three times the upper limit of normal (ULN), and notably, while bilirubin levels can be elevated, they typically stay less than 15 mg/dL [2,6]. Treatment is supportive, and abnormal laboratory values typically resolve within two weeks.

In contrast, AIC is the least common form, with only 17 reported cases [7]. It involves widespread hepatic ischemia from intense sickling, leading to intracanalicular cholestasis. Clinically, patients present with fever and RUQ pain, but laboratory values are often more striking: transaminases may exceed 1,000 IU/L (acute liver failure range), bilirubin can rise above 100 mg/dL, and ALP may be normal or as high as 1,000 IU/L [2,6]. Given its rarity and severity, acute intrahepatic cholestasis may necessitate aggressive measures, including correction of coagulopathy or, in critical circumstances, liver transplantation.

AHS occupies a middle ground between acute sickle cell hepatic crisis and acute intrahepatic cholestasis, occurring in approximately 1.5% of patients with SCD [8]. The pathophysiology of AHS involves the entrapment of sickled erythrocytes within the hepatic sinusoids, where Kupffer cells engulf them. This process results in sinusoidal distention and can lead to compression of the biliary tree. Clinically, AHS presents with acute hepatomegaly, anemia, RUQ pain, and elevated ALP levels, which can reach up to 650 IU/L [2,9,10]. Unlike other hepatic complications of SCD, transaminase levels are typically not markedly elevated [6]. A key laboratory feature of AHS is predominantly conjugated hyperbilirubinemia, with bilirubin levels potentially rising to approximately 24 mg/dL. This pattern is indicative of biliary obstruction rather than extensive hepatocellular injury [2,6].

Although ASCHC, AHS, and AIC within the sickle cell hepatopathy spectrum may share overlapping clinical presentations, distinguishing among them is crucial due to their distinct management strategies. ASCHC generally responds well to supportive care measures such as hydration, analgesia, and addressing the underlying vaso-occlusive crisis. In contrast, AHS often necessitates more aggressive intervention with urgent transfusion support, while AIC may fall either way depending on the clinical presentation, with interventions ranging from simple or exchange transfusion to evaluation for liver transplantation [1,3,11].

In this case, the patient initially presented with leukocytosis, an elevated reticulocyte count, and abnormal liver enzymes, raising suspicion for ASCHC. However, the combination of a marked drop in hemoglobin, extreme hyperbilirubinemia, and imaging findings of significant hepatomegaly indicated AHS as the more likely diagnosis. While her bilirubin level of 35.1 mg/dL did not meet the extreme elevations typically associated with AIC, it significantly exceeded the expected plateau of around 24 mg/dL for AHS, underscoring the severity of her clinical presentation.

Management began with standard treatment for a SCD crisis, including intravenous hydration, pain management, and antibiotic therapy. As her clinical condition deteriorated, treatment was escalated to exchange transfusion. The choice of exchange transfusion over simple transfusion was driven by the need to rapidly reduce the proportion of HbS while minimizing the risk of hyperviscosity from elevated total hemoglobin levels.

Previous case reports of AIC have demonstrated bilirubin elevations ranging from 12.7 mg/dL [12] to as high as 55.1 mg/dL [13]. In the first case, the patient had acute hepatic enlargement that resolved following exchange transfusion [12]. Conversely, in the second case, despite supportive care and transfusions, the patient's condition worsened, necessitating intubation and transfer to a liver transplant center [13]. Another report described a total bilirubin elevation of 35 mg/dL, similar to our patient, complicated by acute hepatitis A infection; however, this patient responded favorably to exchange transfusion and supportive management [14]. Collectively, these cases illustrate that the severity of bilirubin elevation can help guide the intensity of interventions required.

A potential complication of blood transfusions, whether simple or exchange, is "auto-transfusion." This phenomenon occurs when previously sequestered red blood cells are abruptly released back into circulation as sinusoidal obstruction resolves. Such a release can cause a sudden rise in hematocrit and blood viscosity, potentially leading to complications such as cerebrovascular accidents. In these instances, therapeutic phlebotomy may be necessary to reduce the risk of adverse events [15].

## Conclusions

This case highlights the diagnostic and therapeutic complexities inherent to managing SCD with hepatic involvement. Recognizing AHS amidst overlapping features with other forms of sickle cell hepatopathy is challenging but critical. Furthermore, adopting a tailored management approach, including the timely decision to initiate exchange transfusion, can prevent severe complications such as MODS or hyperviscosity. Ultimately, the patient's clinical course underscores the necessity of thorough evaluation and a nuanced, patient-specific strategy in the care of individuals with SCD.
